# Monocyte-Derived Dendritic Cells Are Essential for CD8^+^ T Cell Activation and Antitumor Responses After Local Immunotherapy

**DOI:** 10.3389/fimmu.2015.00584

**Published:** 2015-11-23

**Authors:** Sabine Kuhn, Jianping Yang, Franca Ronchese

**Affiliations:** ^1^Malaghan Institute of Medical Research, Wellington, New Zealand

**Keywords:** dendritic cells, T cells, monocyte-derived dendritic cells, CSF1R, tumor immunotherapy, mouse models

## Abstract

Tumors harbor several populations of dendritic cells (DCs) with the ability to prime tumor-specific T cells. However, these T cells mostly fail to differentiate into armed effectors and are unable to control tumor growth. We have previously shown that treatment with immunostimulatory agents at the tumor site can activate antitumor immune responses and is associated with the appearance of a population of monocyte-derived DCs (moDCs) in the tumor and tumor-draining lymph node (dLN). Here, we use depletion of DCs or monocytes and monocyte transfer to show that these moDCs are critical to the activation of antitumor immune responses. Treatment with the immunostimulatory agents monosodium urate crystals and *Mycobacterium smegmatis* induced the accumulation of monocytes in the dLN, their upregulation of CD11c and MHCII, and expression of iNOS, TNFα, and IL12p40. Blocking monocyte entry into the lymph node and tumor through neutralization of the chemokine CCL2 or inhibition of colony-stimulating factor-1 receptor signaling prevented the generation of moDCs, the infiltration of tumor-specific T cells into the tumor, and antitumor responses. In a reciprocal fashion, monocytes transferred into mice depleted of CD11c^+^ cells were sufficient to rescue CD8^+^ T cell priming in lymph node and delay tumor growth. Thus, monocytes exposed to the appropriate conditions become powerful activators of tumor-specific CD8^+^ T cells and antitumor immunity.

## Introduction

Dendritic cells (DCs) are critical for the induction of adaptive immune responses. DCs in tumors and tumor-draining lymph nodes (dLNs) are often loaded with tumor material and are able to induce proliferation of tumor-specific T cells in the dLN (1–3). Although presentation of tumor antigen by specific DC subsets is necessary for tumor regression in patients and mouse models (4, 5), it is mostly insufficient: proliferating T cells remain in the LN, fail to differentiate into fully armed effector cells, and are unable to infiltrate the tumor and mediate tumor rejection (3).

Several studies have shown that immunostimulating treatments that activate DCs also lead to induction of powerful CD8^+^ T effector cells that eliminate tumor cells and delay tumor growth (6–10). Unexpectedly, by carrying out a detailed study of the effects of these treatments on DC phenotype, we found that successful therapies do not simply activate existing DC subsets, but they also elicit the differentiation of monocytes into monocyte-derived DCs (moDCs) in the dLNs (9, 11, 12). Whether moDCs are simply passengers in the induced inflammatory response or powerful antigen-presenting cells that are especially suited to stimulating potent antitumor immunity remains unclear.

Most of our knowledge of moDCs comes from infection models. Under inflammatory conditions, moDCs can become a substantial population that complements the range of steady state DCs (13–15). MoDCs upregulate CD11c and MHCII but generally retain expression of monocyte markers such as Ly6C, Ly6B, CD64, and FcϵRIα (15–17). They can mediate effector functions via their production of the Th1 cytokines TNFα and IL-12 as well as through direct cytotoxicity via NO production (13, 15, 17, 18). Importantly, moDCs can also prime Th1 immunity and cytotoxic T cell responses (14, 15, 19), recognized components of successful antitumor responses (4, 20, 21).

In a tumor context, the role of monocytes as precursors of immunostimulatory moDCs is rarely considered. Instead, Ly6C^hi^ CCR2^+^ monocytes are known to give rise to the majority of tumor-associated macrophages (22–24) and contribute to the pool of monocytic myeloid-derived suppressor cells (MDSCs) that promote tumor growth and suppress antitumor immunity (25, 26). Nonetheless, experiments in mouse models show that monocytes can also differentiate into inflammatory DCs that produce reactive oxygen species (ROS) and directly mediate antitumor responses independently of effector T cells (27). Similarly, in human skin lesions treated with toll-like receptor 7/8 agonists, inflammatory DCs acquire expression of cytotoxic mediators *in vivo* and become directly tumoricidal *in vitro* (28).

In addition to acquiring direct tumoricidal function, moDCs can also serve as antigen-presenting cells in the tumor context. In a recent study, anthracyclin chemotherapy induced Ly6C^hi^ CD11c^+^ cells at the tumor site by an ATP- and CCR2/CCL2-dependent mechanism (29, 30). These cells, but not PDCA^+^ pDCs or BATF3-dependent DCs, could activate T cells *in situ* and were necessary for antitumor activity (29). Interestingly, lymph nodes or tertiary lymphoid tissues were not necessary for this response, suggesting that it might rely on existing memory T cells rather than *de novo* priming of naive T cells. It remains to be determined whether the key role of moDCs is specific to chemotherapy, or whether it may extend to antitumor immune responses induced by other treatments. In this respect, it is noteworthy that work from our group indicates that moDCs can also be elicited by peritumoral treatment with the toll-like receptor 3 ligand polyI:C, or the immunostimulatory agents monosodium urate (MSU) crystals and *Mycobacterium smegmatis* (*Msmeg*). Importantly, we find that the presence of moDCs correlates with CD8^+^ T cell activation and treatment success in several tumor models (9, 12). Whereas moDCs are frequently observed after exposure to infectious stimuli, the precise conditions leading to their generation have not been determined. In our model, polyI:C and MSU + *Msmeg* both induced elevated levels of IFNγ and IL-12p70 in serum (9). These cytokines were reported to play a key role in moDC generation and function (18). In addition, we observed that MSU + *Msmeg* induced the release of IL-1β, which was required for the antitumor response (11).

In this paper, we sought to determine the relevance of moDCs in antitumor immunity. We used a model of murine melanoma and local treatment with MSU + *Msmeg* to show that moDCs are critical for treatment success. MSU + *Msmeg* induced recruitment of monocytes from blood and their differentiation into inflammatory moDCs in the dLN. Treatment with a colony-stimulating factor-1 receptor (CSF1R) inhibitor similar to the ones currently in clinical trial to block MDSCs also blocked monocyte and moDC accumulation in the dLN, as well as tumor-specific T cell proliferation in dLN and antitumor activity. Finally, adoptively transferred monocytes were able to differentiate into CD11c^+^ moDCs *in vivo* and were sufficient to restore MSU + *Msmeg* antitumor responses in CD11c-depleted animals. Taken together, these results indicate that moDCs are critical for the success of MSU + *Msmeg* immunotherapy and suggest a common mechanism by which immunotherapy and chemotherapy may be able to transform tumors into sites of immune activation.

## Materials and Methods

### Mice

All mice were bred at the Malaghan Institute of Medical Research Biomedical Research Unit. C57BL/6J (CD45.2^+^), B6.SJL-Ptrprc^a^ (CD45.1^+^), and CD11c-DTR mice were originally from Jackson Laboratories, USA; OTI mice expressing a transgenic TCR specific for K^b^ + ovalbumin (OVA)_257–264_ were from Melbourne University, Australia. CD11c-DTR bone marrow (BM) chimeras were generated as described (9) by irradiating (2 × 550 rad) C57BL/6J hosts followed by i.v. transfer of 10^7^ CD11c-DTR BM cells. Chimeras were rested for at least 8 weeks before being used in experiment. All experimental procedures were approved by the Victoria University of Wellington Animal Ethics Committee.

### Tumor Cell Lines and Tumor Challenge

The B16-F1 murine melanoma (American Type Culture Collection, ATCC) and the B16.OVA melanoma expressing a truncated OVA protein (31) were maintained in complete Iscove’s modified Dulbecco’s medium as described (1), and extended *in vitro* passaging was avoided. For tumor challenge, cells were washed 3× in medium, and 10^5^ tumor cells were injected s.c. into the flank of mice. Tumor size and survival were calculated as described (1).

### MSU + *Msmeg* Treatment

Mice were treated with 2 × 10^6^ colony-forming units (CFU) of *Msmeg* (mc^2^155) and 250 μg MSU crystals (containing <0.01 EU/10 mg) in a total volume of 100 μl PBS as described (9). Full treatment involved four injections of MSU + *Msmeg* given s.c. around the tumor, starting when the tumor became palpable (usually around days 7–9) and repeated every second day thereafter. Control mice received 100 μl PBS (Invitrogen).

### Flow Cytometry

Lymph nodes or tumors were digested using DNase I and Liberase TL (Roche) as described (9). Blood was collected in Alsever’s solution, and red blood cells were lysed in ammonium-chloride-tris buffer (both made in house). Single cell suspensions were then resuspended in FACS buffer (PBS with 10 mM EDTA, Sigma; 2% FBS, Gibco; and 0.01% NaN_3_, Sigma) and blocked with anti-mouse CD16/32 (2.4G2) before staining with fluorescent antibodies specific for the following markers: CD45 (30F11), CD45.1 (A20), CD4 (GK1.5), CD8α (53-6.7), CD11c (HL3), CD11b (M1/70), Vα2 (B20.1), and Vβ5.1/5.2 (MR9-4), all from BD Biosciences; Ly6C (HK1.4), CD64 (X54-5/7.1), CD135 (A2F10), and MHCII (M5/114.15.2) from Biolegend; CD115 (AFS98), CD45.2 (104), and Ly6G (1A8) from eBioscience; Ly6B (7/4) from AbD Serotec; and CCR2 (475301) from R&D Systems. Anti-CD4 (GK1.5), -CD8α (2.43), and -MHCII (3JP) were affinity purified from hybridoma supernatants. Streptavidin-PE, -APC, or PE-Texas-Red (BD Biosciences) were used where required. Dead cells were excluded by staining with 6′-diamidino-2-phenylindole (DAPI) or Live/Dead Fixable blue (Invitrogen). For intracellular cytokine staining, cell suspensions were incubated for 6 h in 1 μg/ml Golgi Stop (BD Biosciences) and 2 μg/ml Brefeldin A (eBioscience) with no restimulation. After surface staining, cells were stained with anti-IL-12p40 (C17.8 from eBiosciences), anti-TNF-α (MP6-XT22, from BD Biosciences) antibodies, or isotype controls (R3-34 BD Biosciences or EBRG1, eBioscience), using the BD Cytofix/Cytoperm kit (BD Biosciences). Staining for iNOS was done in the same way with M-19 polyclonal antibody (sc-650, Santa Cruz Biotechnology) or control rabbit IgG (sc-2027, Santa Cruz Biotechnology) but without incubation with Golgi Stop/Brefeldin A. Acquisition was performed on a BD LSRII SORP or a BD LSR Fortessa SORP (Becton Dickinson), and data were analyzed using FlowJo Version 9.8.3 (Tree Star).

### DC Depletion

For DC depletion, CD11c-DTR BM chimeras were injected i.p. with 15 ng diphtheria toxin (DT) (Sigma-Aldrich) per gram body weight 12 h prior to each MSU + *Msmeg* treatment. In some experiments, depletion was confirmed by flow cytometry of tumor-draining LNs and tumors as shown in Figure S1 in Supplementary Material.

### *In vivo* T Cell Proliferation

CD8^+^ T cells from spleens and LNs of OTI × B6.SJL-Ptprca mice were positively selected using anti-CD8^+^ MACS Beads (Miltenyi Biotec) and labeled with CFSE as described (32). Purity was >87% as assessed by positive staining with anti-CD8α, -Vα2, and -Vβ5 antibodies. About 2 × 10^6^ cells were injected i.v. into C57BL/6J or CD11c-DTR BM chimeric hosts bearing established B16.OVA tumors. OTI proliferation was assessed in tumor-draining LNs 6 days later.

### Inhibition of CSF1 Signaling

The small-molecule GW2580, a selective inhibitor of the CSF1 receptor kinase (33), was used to block CSF1 signaling *in vivo*. Tumor-bearing mice received six daily doses of either 160 mg/kg GW2580 (kindly provided by Prof Peter Shepherd, University of Auckland, New Zealand) in a solution of 0.5% methylcellulose (Sigma) and 0.1% Tween80 (Sigma) in dH_2_O, or control diluent, by oral gavage starting 4 h before the first MSU + *Msmeg* treatment. This dose was previously shown to achieve 100% inhibition of CSF1-dependent monocyte growth (33).

### Monocyte Isolation from BM and Adoptive Transfer

Monocytes were enriched from BM as described (15, 34) with minor modifications. BM cell suspensions were incubated with biotinylated antibodies specific for the following markers: B220 (RA3-6B2), CD19 (1D3), CD49b (DX5), CD90.2 (53-2.1), and Ly-76 (Ter119) (all from eBioscience); Ly6G (1A8) and CD135 (A2F10) (from Biolegend); and CD11c (HL3) and CD24 (M1/69) (from BD Biosciences), followed by incubation with anti-Biotin MACS beads (1 μl/10^6^ cells) and magnetic separation on an Automacs (Miltenyi Biotec). The enriched population contained 85–91% Ly6C^high^ monocytes, the majority of which also expressed CD11b, Ly6B, CD64, CCR2, and CD115 (CSF1R). Recipients received 1–2 × 10^6^ enriched monocytes i.v.

### CCL2 Blocking

Mice bearing palpable B16.OVA tumors were injected i.p. with 100 μg anti-CCL2 antibody (clone 2H5, Biolegend) or Armenian hamster IgG control antibody (clone HTK888, Biolegend) 2 h prior to i.v. transfer of purified BM monocytes.

### Statistical Analyses

Statistical analyses were done using GraphPad Prism 5. Two-tailed Student’s *t*-test was used to compare two groups. Multiple groups were compared by one-way ANOVA with Tukey’s post-test, or Kruskal-Wallis test with Dunn’s post-test for data that were not normally distributed. Survival was analyzed using the log-rank test with Bonferroni’s correction for multiple testing. Differences of *p* < 0.05 were deemed significant (*), *p* < 0.01 very significant (**), and *p* < 0.001 extremely significant (***).

## Results

### DCs Are Required for Tumor Immunotherapy by MSU + *Msmeg*

**Figure 1:**
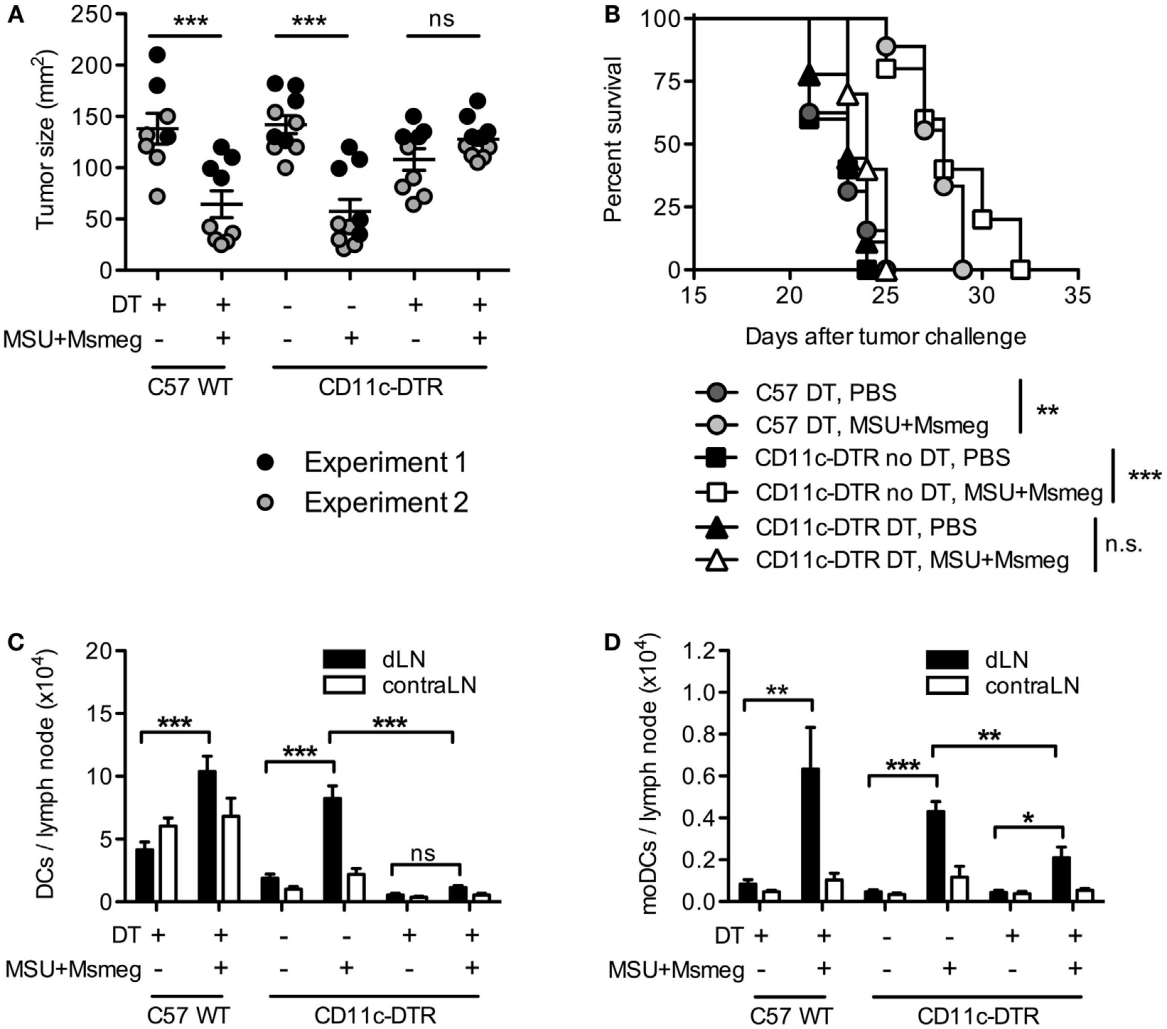
**Dendritic cells are required for the activation of antitumor immunity by MSU + *Msmeg***. C57BL/6 WT (C57WT) or CD11c-DTR BM chimeras were injected with B16 melanoma tumors, treated every second day for four times with MSU + *Msmeg* or PBS, and at the same time depleted of CD11c^+^ cells by i.p. DT treatment as indicated. **(A)** Individual tumor sizes in each group, as measured on the day when average tumor size in the C57 WT control group approached 150 mm^2^ (days 17–23 after tumor challenge). Horizontal lines show mean ± SEM. **(B)** Survival of tumor challenged mice. Data in **(A,B)** are pooled from two independent experiments each with 3–5 mice/group. **(C)** Numbers of total DCs (CD11c^+^MHCII^+^) and **(D)** numbers of moDCs (CD11c^+^MHCII^+^CD11b^+^CD64^+^Ly6C^+^) in draining and contralateral LNs 2 days after the completion of MSU + *Msmeg* treatment. Data refer to one of two independent experiments that gave similar results. Graphs show mean + SEM for 5 mice/group. Statistical analysis in **(A,C,D)** was by one-way ANOVA with Tukey’s post-test. **(B)** Log-rank test with Bonferroni’s correction for multiple testing.

Treatment of established B16 melanomas with the immune-activating agents MSU + *Msmeg* delays tumor growth and increases survival (9, 11). Treatment success requires adaptive immunity, in particular CD8^+^ T cells, and correlates with the accumulation of moDCs in dLN as early as 1 day after the first MSU + *Msmeg* treatment (9). To test whether DCs are required for MSU + *Msmeg* immunotherapy, we used CD11c-DTR BM chimeras and depleted them of CD11c^+^ cells by administration of DT throughout the duration of MSU + *Msmeg* treatment. Depletion of CD11c^+^ cells abrogated the treatment-induced reduction in tumor size and increase in survival (Figures 1A,B). Successful depletion of CD11c^+^MHCII^+^ DCs in dLN after the last treatment was verified in separate experiments (Figure 1C) and confirmed that different DCs subsets, including CD8α^+^ DCs, CD11b^+^ DCs, CD8α^−^CD11b^−^ DCs, and CD11b^+^CD64^+^Ly6C^+^ moDCs, were all significantly reduced in DT-treated CD11c-DTR chimeras (Figure 1D; Figure S1 in Supplementary Material). Thus, CD11c^+^ DCs are necessary for MSU + *Msmeg* antitumor activity.

### MSU + *Msmeg* Recruits Monocytes from Blood to dLN and Induces Expression of DC Markers

**Figure 2:**
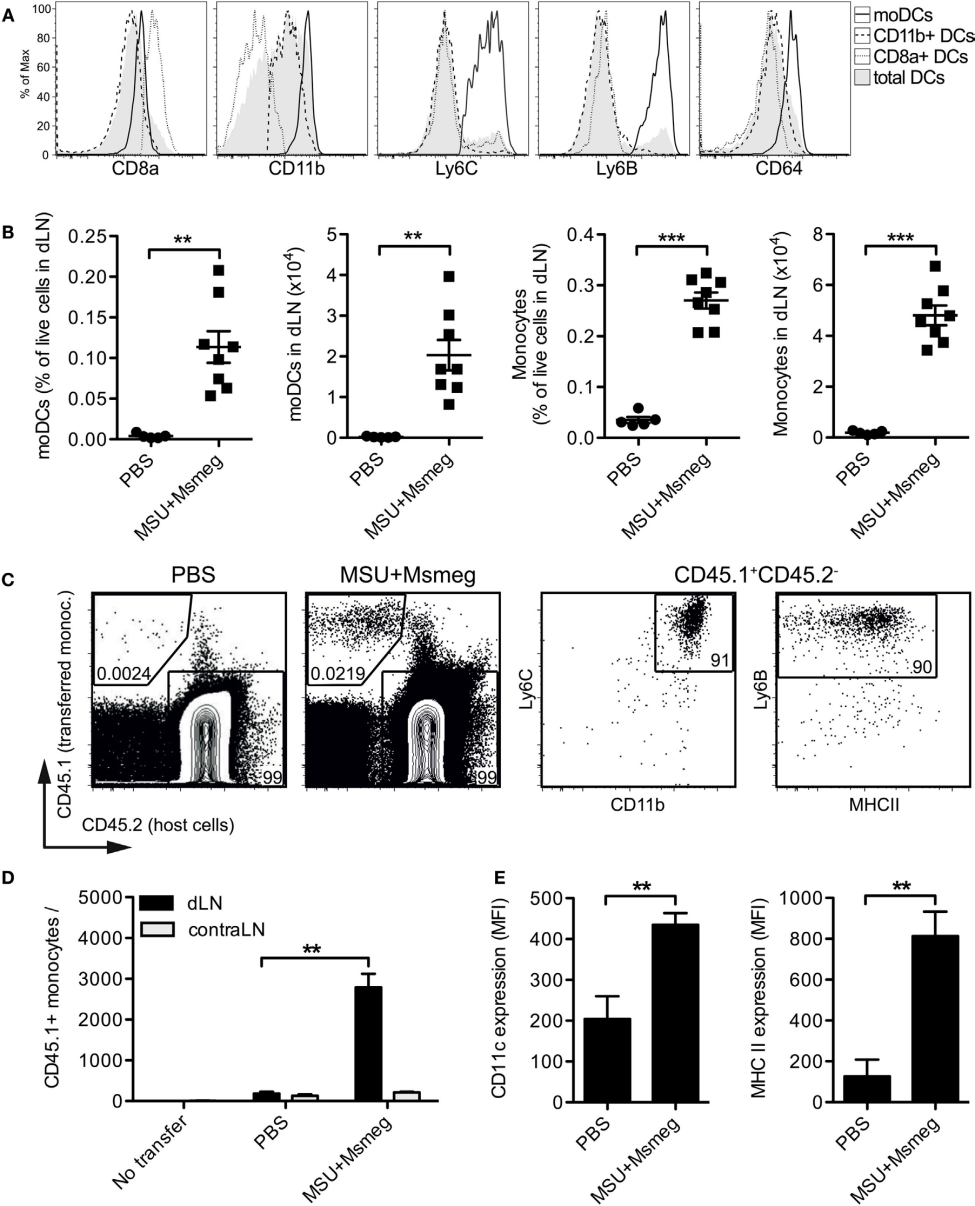
**Monocyte accumulation and upregulation of DCs markers in the dLN of MSU + *Msmeg*-treated mice**. **(A,B)** Mice were injected s.c. with MSU + *Msmeg* or PBS on days 0 and 2. About 17 h later dLNs were harvested and analyzed by flow cytometry. **(A)** Expression of DC and monocyte lineage markers on total DCs (MHCII^+^CD11c^+^), CD11b^+^ DC (CD11b^+^CD8^−^Ly6B^−^Ly6C^−^ DCs), CD8a^+^ DC (CD8a^+^CD11b^−^ DCs), and moDCs (CD11b^+^Ly6B^+^Ly6C^+^ DCs). **(B)** Frequency and number of moDCs (CD11b^+^Ly6B^+^Ly6C^+^CD11c^+^MHCII^+^) and monocytes (CD11b^+^Ly6B^+^Ly6C^+^CD11c^−^) in dLN. Data are pooled from two independent experiments each with 3–5 mice/group. **(C,D,E)** As in **(A)** but mice received two million CD45.1^+^ BM monocytes i.v. 17 h before analysis. **(C)** Identification of transferred monocytes in dLN. **(D)** Number of transferred monocytes and **(E)** expression of CD11c and MHCII on transferred monocytes, expressed as median fluorescence intensity (MFI). Data in **(C–E)** are from one of two independent experiments, each with 3–5 mice/group, that gave similar results. All graphs show mean + SEM. Statistical analyses used a Student’s *t*-test.

We sought to establish the origin of the moDCs induced by MSU + *Msmeg* treatment. A substantial population of moDCs was observed in the dLN of intact mice that received MSU + *Msmeg* subcutaneously. MoDCs formed a distinct population within the CD11c^+^MHCII^+^ DCs, uniformly expressing high levels of CD11b, Ly6C, Ly6B, and CD64 (Figure 2A). The numbers of moDCs and monocytes in dLN were comparable to the numbers seen in similarly treated tumor-bearing animals [Figure 2B (9)] and represented about 10% of the total DC population in treated dLNs. In contrast, the proportions of other DC subsets were not significantly affected by MSU + *Msmeg* treatment (9).

We then examined the fate of purified BM monocytes transferred into the blood of recipient mice. Transferred cells were recruited to MSU + *Msmeg*-treated dLN (Figures 2C,D), and by 17 h after transfer they had already upregulated expression of the DC markers CD11c and MHCII (Figure 2E). Thus, the early influx of moDCs into the dLN of MSU + *Msmeg*-treated mice is likely due to monocytes entering the dLN directly from the blood, rather than monocytes migrating from the tumor to the dLN. However, these data do not rule out that, at later time points, moDCs in dLN may originate from either tumor or blood.

### CCR2^+^ Monocytes Enter dLN and Tumors in a CCL2-Dependent Manner

**Figure 3:**
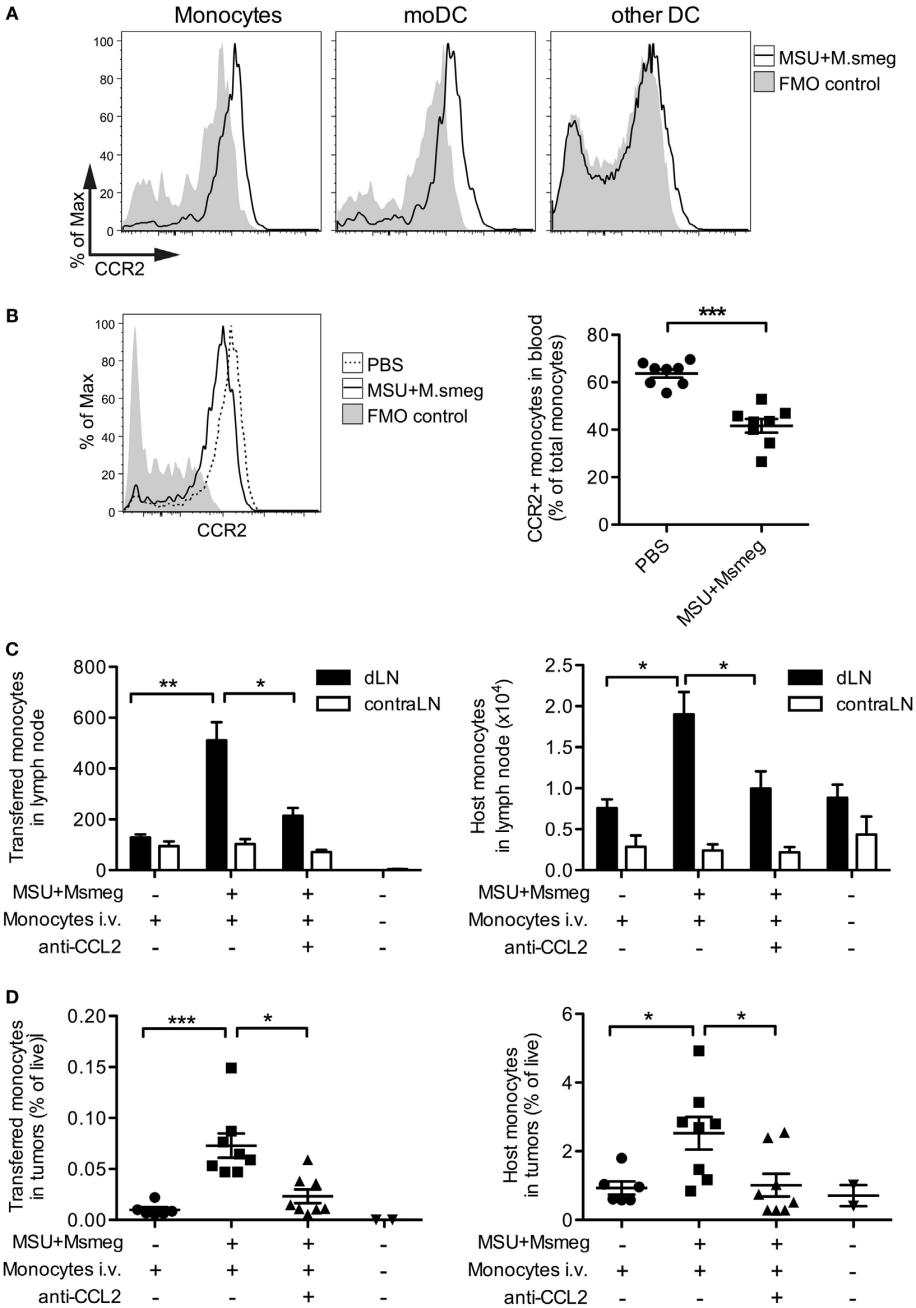
**CCL2 is necessary for the recruitment of monocytes to MSU + *Msmeg*-treated dLNs and tumors**. **(A)** Expression of CCR2 on monocytes (CD11b^+^Ly6B^+^Ly6C^+^CD11c^−^), moDCs (CD11c^+^MHCII^+^Ly6B^+^Ly6C^+^), and other DCs (CD11c^+^MHCII^+^Ly6B^−^Ly6C^−^) in dLNs of mice treated with MSU + *Msmeg* once 45 h earlier. **(B)** Frequency of CCR2^+^ monocytes in blood 15 h after MSU + *Msmeg* treatment. **(C,D)** Mice bearing palpable B16.OVA tumors were treated with anti-CCL2 or control antibody. About 2 h later, mice received 2 million purified CD45.1^+^ naive monocytes and were treated with MSU + *Msmeg*. The recruitment of transferred (CD45.1^+^) or host (CD45.2^+^) monocytes (Ly6B^+^Ly6C^+^CD11c^−^) to LN **(C)** and tumors **(D)** was examined 18 h later. Data are pooled from two independent experiments each with 3–5 mice/group. All graphs show mean + SEM. Statistical analyses used a Student’s *t*-test **(B)** or a Kruskal–Wallis with Dunn’s post-test **(C,D)**.

Blood monocytes can be divided into two subsets: Ly6C^low^ patrolling monocytes that migrate in and out of tissues in the steady state, and Ly6C^high^ inflammatory monocytes that express CCR2 and are actively recruited to sites of inflammation (35). We therefore examined CCR2 expression on monocytes, moDCs, and cDCs in the dLN of mice treated with MSU + *Msmeg* 45 h earlier. Monocytes and moDCs both expressed CCR2, while other Ly6B^−^Ly6C^−^ DCs were mostly CCR2 negative (Figure 3A). Consistent with this observation, the frequency of blood monocytes expressing CCR2^+^ was diminished 15 h after MSU + *Msmeg* treatment, suggesting recruitment into inflamed tissues (Figure 3B).

We also investigated the role of CCL2 in the recruitment of monocytes to the dLN. CCL2, also known as MCP-1, is the main ligand of CCR2, but its role in the migration of monocytes to sites of inflammation is controversial (19, 36). Systemic treatment with CCL2-blocking antibodies almost completely inhibited the recruitment of both i.v. transferred BM monocytes and host monocytes to the dLN (Figure 3C). The proportions of transferred and host monocytes in tumors were also significantly reduced in anti-CCL2-treated mice (Figure 3D). The effects of CCL2 blockade on tumor progression were not determined. Thus, CCR2^+^ monocytes enter the dLN and tumors of MSU + *Msmeg*-treated mice in a CCL2-dependent manner.

### Blocking Monocyte and moDC Recruitment Abolishes MSU + *Msmeg*-Induced Antitumor Activity

To begin to address the contribution of moDCs to the antitumor immune response induced by MSU + *Msmeg*, we used treatment with GW2580, a small-molecule inhibitor highly specific for the CSF1R kinase (33). CSF1 promotes proliferation, survival, and differentiation of monocytes, and blocking CSF1R signaling impairs monocyte recruitment without affecting conventional DC subsets [Ref. (37) and data not shown].

**Figure 4:**
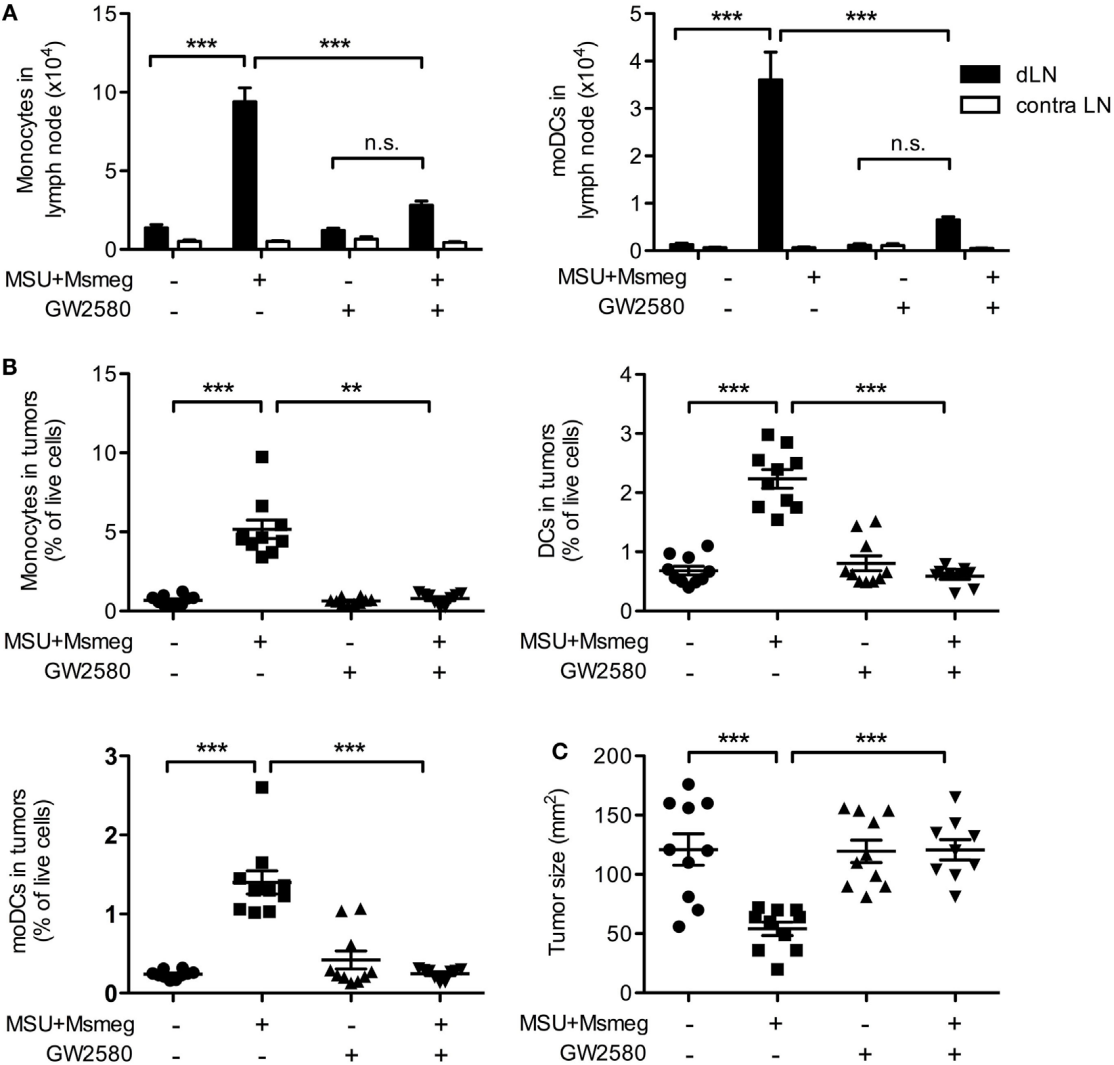
**Treatment with the CSF1-signaling inhibitor GW2580 impairs monocyte and moDCs recruitment and abrogates the antitumor activity of MSU + *Msmeg***. Mice bearing B16.OVA tumors were treated with MSU + *Msmeg*, and at the same time they received daily oral doses of GW2580 or vehicle control. LN and tumors were analyzed 2 days after the last MSU + *Msmeg* treatment. **(A)** Numbers of monocytes (CD11b^+^Ly6B^+^Ly6C^+^CD64^+^CD11c^−^) and moDCs (CD11b^+^Ly6B^+^Ly6C^+^CD11c^+^MHCII^+^) in dLN. **(B)** Frequencies of monocytes (CD45^+^CD11b^+^Ly6C^high^Ly6G^−^CD11c^−^), total DCs (CD45^+^CD11c^+^MHCII^+^), and moDCs (CD45^+^CD11c^+^MHCII^+^CD11b^+^Ly6C^+^Ly6B^+^) in tumors. **(C)** Tumor sizes on day 13. All data are pooled from two independent experiments each with 5 mice/group. Graphs show mean + SEM. Statistical analyses used ANOVA with Tukey’s post-test **(A)** or a Kruskal–Wallis with Dunn’s post-test **(B,C)**.

Treatment with GW2580 induced a marked reduction in monocytes and moDCs in the dLNs (Figure 4A) and tumors (Figure 4B and data not shown) of MSU + *Msmeg*-treated mice. In addition, administration of GW2580 to prevent monocyte and moDC recruitment throughout the duration of MSU + *Msmeg* treatment completely abrogated the antitumor effect (Figure 4C). We therefore conclude that moDCs are required for MSU + *Msmeg* tumor therapy.

### Monocytes and moDCs Produce Proinflammatory Factors and Are Necessary for Proliferation and Tumor Infiltration of CD8^+^ T Cells

**Figure 5:**
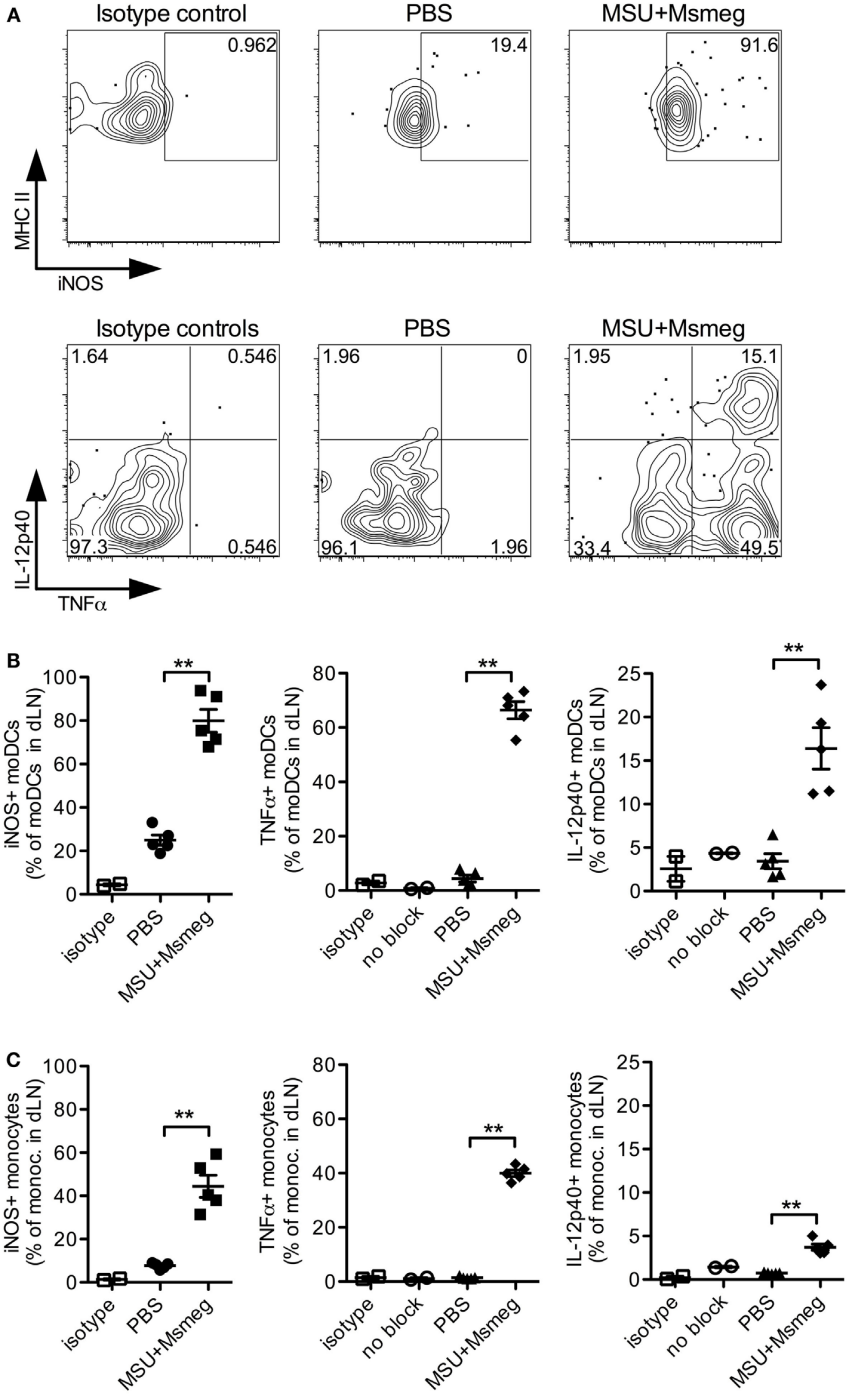
**Monocytes and moDCs in dLN express proinflammatory mediators after MSU + *Msmeg* treatment**. Mice were treated with a single dose of MSU + *Msmeg* s.c., and 19 h later dLNs were collected and analyzed by intracellular staining for the indicated markers. Staining controls included an isotype control (isotype) and stained samples that were not treated with GolgiStop/Brefeldin A (no block). **(A)** Representative flow plots and **(B)** frequencies of iNOS-, IL-12-, and TNFα-expressing moDCs (CD11c^+^MHCII^+^CD11b^+^Ly6B^+^Ly6C^+^). **(C)** Frequencies of monocytes (CD11b^+^Ly6B^+^Ly6C^+^CD11c^−^) expressing proinflammatory molecules. Data are from one of two independent experiments, each with 3–5 mice/group that gave similar results. Graphs show means + SEM. Statistical analyses used a Student’s *t*-test.

To elucidate the function of moDCs in mediating the antitumor activity of MSU + *Msmeg*, we chose to first investigate whether they produce proinflammatory cytokines and iNOS. During infections, moDCs are well known to be major producers of TNFα and iNOS (13) and can also be a critical source of IL-12 (15, 18). In MSU + *Msmeg*-treated mice, the majority of moDCs in dLN were positive for intracellular iNOS and TNFα (Figures 5A,B). In addition, about 15% of moDCs costained for both intracellular TNFα and IL-12p40 (Figures 5A,B, right panel). Monocytes in the same dLN showed a similar pattern of intracellular iNOS, TNFα, and IL-12p40 staining, but the proportion of cytokine-expressing cells was lower compared to moDCs (Figure 5C).

**Figure 6:**
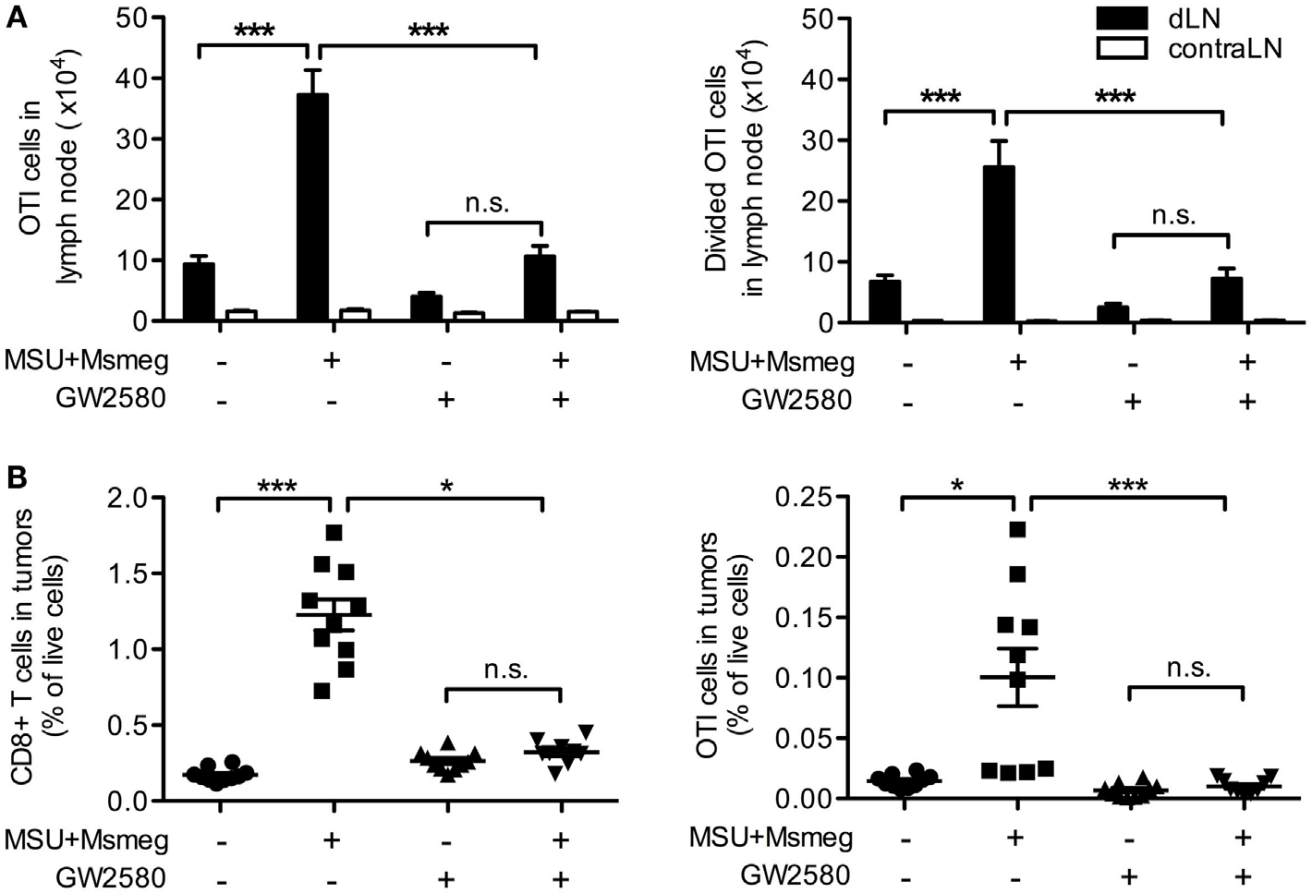
**Monocyte-derived DCs are necessary for the proliferation of tumor-specific CD8^+^ T cells in dLN and their infiltration into tumors**. Mice bearing B16.OVA tumors were treated with MSU + *Msmeg*, and at the same time they were given daily oral doses of GW2580 or vehicle. LN and tumors were analyzed 2 days after the last MSU + *Msmeg* treatment. **(A)** Numbers of total and divided OTI T cells (CD45.1^+^CD8α^+^ lymphocytes) in tumor dLN, as assessed by CFSE dilution. **(B)** Total CD8^+^ and OTI T cells (CD45^+^CD8α^+^ and CD45^+^CD8α^+^CD45.1^+^, respectively) expressed as frequencies of total live cells in tumors. Data are pooled from two independent experiments, each with 5 mice/group. All graphs show mean + SEM. Statistical analyses were by one-way ANOVA with Tukey’s post-test **(A)** and Kruskal–Wallis test with Dunn’s post-test **(B)**.

Monocyte-derived DCs have been reported to crossprime CD8^+^ T cells (38, 39) and induce strong Th1 and cytotoxic T lymphocyte responses (14, 15, 19). We used B16 tumors expressing the model antigen OVA together with adoptive transfer of CFSE-labeled naive CD45.1^+^ OTI T cells to assess priming of tumor-specific CD8^+^ T cells in response to MSU + *Msmeg*. Consistent with our previous work (9), MSU + *Msmeg* treatment increased the number of total and divided OTI cells in dLN (Figure 6A). Reducing the number of moDCs in dLN by treatment with GW2580 inhibited the increase in OTI proliferation (Figure 6A), indicating that moDCs were necessary for this response. We then examined the percentage of CD8^+^ T cells and OTI cells in tumors. Again, GW2580 fully abrogated the increase in CD8^+^ T cell and OTI cell infiltration into MSU + *Msmeg*-treated tumors (Figure 6B). These data indicate that moDCs are necessary for the efficient proliferation of tumor-specific T cells in the dLN of MSU + *Msmeg*-treated mice, and for their accumulation in the tumor.

### moDCs Are Sufficient for MSU + *Msmeg* Antitumor Activity

**Figure 7:**
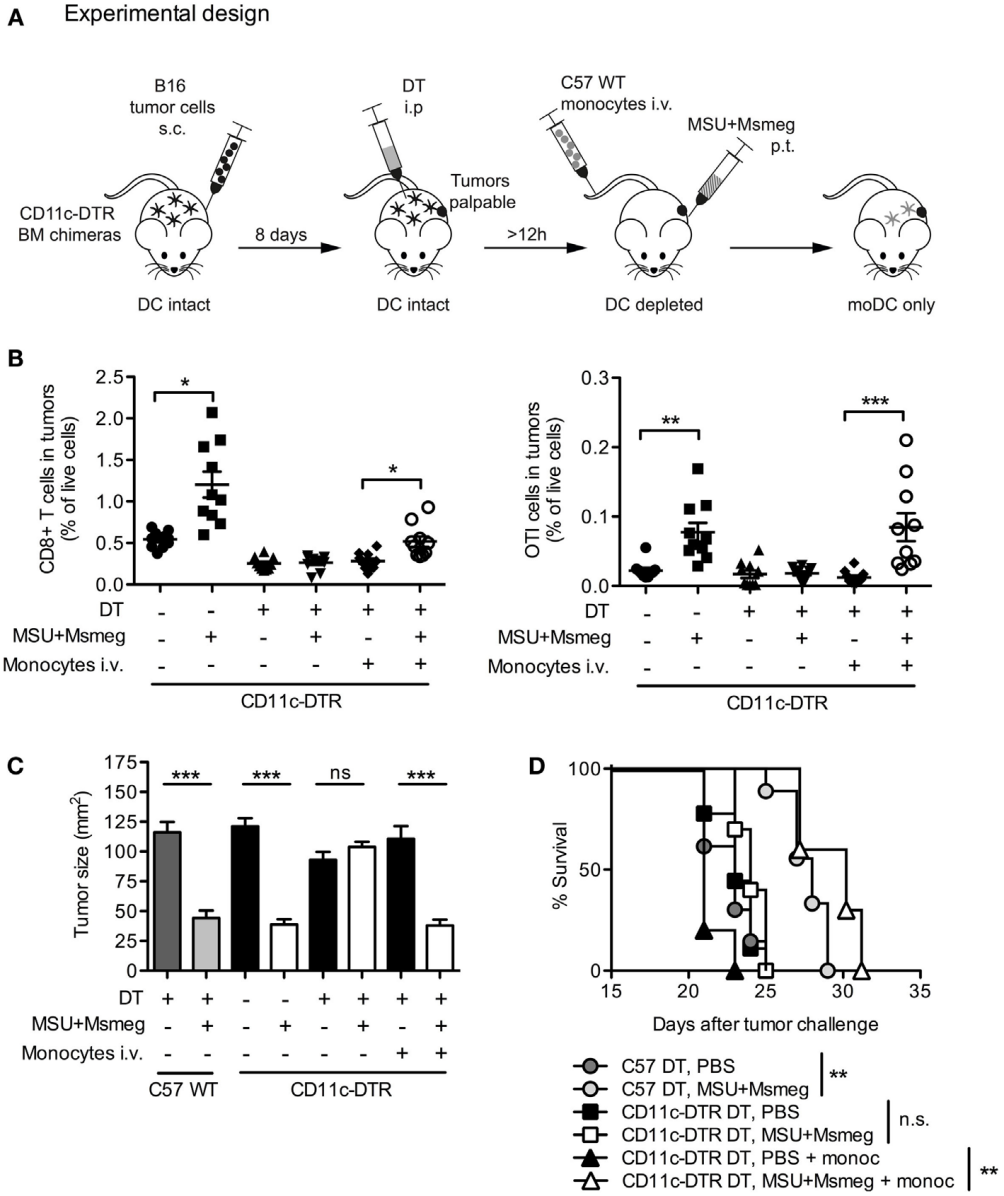
**Monocyte-derived DCs are sufficient for the recruitment of CD8^+^ T cells to tumors and the activation of antitumor immunity**. **(A)** Schematic representation of experimental design. C57BL/6 WT (C57WT) or CD11c-DTR BM chimeras were injected with B16 melanoma tumors, treated every second day for four times with MSU + *Msmeg* or PBS, and at the same time depleted of CD11c^+^ cells by DT treatment as indicated. Some of these mice were also injected with WT BM monocytes to obtain mice where moDC populations could only originate from the injected monocytes. **(B)** Mice were treated as in **(A)**, except that tumors were B16.OVA, and all mice also received naive OTI cells given at the time of the second dose of immunotherapy. Graphs show the percentage of CD8^+^ T cells (CD45^+^CD4^−^CD8^+^) and OTI cells (CD45^+^CD4^−^CD8^+^CD45.1^+^) among total live cells in tumors 2 days after the fourth MSU + *Msmeg* treatment. Data are pooled from two independent experiments each with 5 mice/group. Graphs show mean + SEM. Statistical analysis used a Kruskal–Wallis test with Dunn’s post-test. **(C)** Mean tumor sizes + SEM on day 21 after tumor challenge. Data are pooled from three independent experiments each with 5 mice/group. Statistical analysis was by ANOVA with Tukey’s post-test. **(D)** Survival data, pooled from two independent experiments each with 5 mice/group. Statistical significance was evaluated using a log-rank test and Bonferroni’s correction.

To assess whether moDCs are sufficient for the development of antitumor immunity after MSU + *Msmeg* treatment, we used an experimental setup similar to the one outlined in Figure 7A to generate mice in which moDCs were the only DC subset present. Tumor-bearing CD11c-DTR BM chimeric mice were injected with DT to deplete CD11c^+^ cells throughout the duration of MSU + *Msmeg* treatment. At the same time, mice were adoptively transferred with BM monocytes from wild-type (WT) C57BL/6 mice. As WT monocytes cannot express DTR, they were not affected by DT treatment and could differentiate into CD11c^+^ moDC when exposed to the appropriate conditions. In some experiments, chimeras were also injected with B16.OVA tumors and adoptively transferred with naive OTI CD8^+^ T cells to assess the response of tumor-specific CD8^+^ T cells.

B16.OVA tumors were analyzed for percentage of total CD8^+^ T cells and antigen-specific OTI cells at the end of MSU + *Msmeg* treatment. Depletion of CD11c^+^ cells at the time of MSU + *Msmeg* treatment abrogated the increase in CD8^+^ T cell infiltration into the tumor. Transfer of WT monocytes into CD11c-depleted mice was sufficient to partially recover CD8^+^ T cell accumulation (Figure 7B, left panel) and completely restored the frequency of OTI T cells to levels comparable to those in CD11c-sufficient controls (Figure 7B, right panel). Transfer of monocytes also restored the antitumor activity of MSU + *Msmeg*, resulting in reduced tumor size (Figure 7C) and increased survival similar to WT controls (Figure 7D). Recovery of the antitumor response did not require transfer of OTI T cells and occurred regardless of challenge with B16 or B16.OVA tumors, or OTI transfer. These results indicate that, in a situation where other CD11c^+^ cells are depleted, moDCs are sufficient for induction of tumor-specific CD8^+^ T cell responses and successful tumor immunity in response to MSU + *Msmeg*.

## Discussion

In this study, we investigated the role of moDCs in the antitumor immune response elicited by local treatment with MSU + *Msmeg*. We show that DCs are necessary for the activity of MSU + *Msmeg*, as depletion of CD11c^+^ cells completely abrogated the antitumor effect. Interestingly, blockade of CSF1R to selectively deplete moDCs without affecting other DC subsets also abrogated the effect of immunotherapy. In a reciprocal fashion, adoptive transfer of monocytes into mice depleted of CD11c^+^ cells was sufficient to restore antitumor activity. MoDCs originated from circulating monocytes in response to MSU + *Msmeg* treatment, expressed iNOS, TNFα, and IL-12 and were necessary for the priming of tumor-specific CD8^+^ T cells. In addition, moDCs were necessary and sufficient for the accumulation of tumor-specific CD8^+^ T cells in tumors. Together, these data show that moDCs play an essential role during immunotherapy with MSU + *Msmeg*.

The experiments in this paper investigated the role of moDCs using a model of B16 melanoma and immunotherapy with MSU + *Msmeg*. Our previous work compared several different treatments with immunostimulatory agents, including LPS, polyI:C (9), and CpG immunostimulatory oligonucleotides (unpublished data), to show that antitumor activity correlated with increased numbers of moDCs in the tumor dLN. In addition, treatments that were effective on B16 melanoma were also effective on EL4 thymomas (11) and 4T1 mammary carcinomas (9), suggesting that the requirement for moDCs in antitumor immune responses extends to other forms of immunotherapy besides MSU + *Msmeg*, and to multiple tumor models.

Our finding that CCR2^+^Ly6C^high^ monocytes were recruited to MSU + *Msmeg* dLNs and tumors is consistent with literature showing that these monocytes are preferentially attracted to sites of inflammation (35). Accordingly, the strong reduction in monocyte recruitment after treatment with CCL2-blocking antibodies suggests that CCR2–CCL2 interactions are largely responsible for the attraction of monocytes to the site of MSU + *Msmeg* treatment (23, 30, 36). While there is clear evidence that monocytes can differentiate into moDCs during inflammation (13, 14, 35) surprisingly little is known about the signals that govern this process. In line with our findings, CSF1 is the major lineage regulator of mononuclear phagocytes in the BM and thus is required for the development of monocytes and subsequently moDCs (40). The upregulation of MHCII and production of IL-12p40 by moDCs instead were shown to depend on natural killer (NK) cell-derived IFNγ in a study of *Toxoplasma gondii* infection (18). In our model, depletion of NK1.1^+^ cells did not reduce the number of MHCII^+^ moDCs in dLN after MSU + *Msmeg* treatment (data not shown), suggesting that NK cells were not vital for moDC differentiation. However, NK cells may be important for the function of moDCs, a possibility that was not examined in our experiments.

In our study, a significant proportion of moDCs expressed IL-12p40. The critical role of IL-12 for the induction of cytotoxic T lymphocytes and for antitumor immunity is well established. In addition, moDCs produced TNFα and iNOS. This activity was previously used as a defining feature of monocyte-derived “TIP” DCs (13). Mounting evidence suggests that TNFα is important for antitumor immunity, as shown by experiments where tumor immunotherapy with polyI:C required TNFα for treatment success (41). Furthermore, a recent study showed that the combined action of TNFα and IFNγ is necessary for the control of a range of murine and human tumors by driving cancer cells into senescence (20).

In contrast to IL-12 and TNFα, the role of NO/iNOS in tumors is more controversial. A number of studies have shown that macrophage- or MDSC-derived NO can suppress antitumor T cell responses *in vitro* [reviewed in Ref, (42)]. However, others report that NO is required for direct tumoricidal activity of macrophages *in vitro* (43, 44). *In vivo* data are unfortunately limited and suggest that the effects of NO *in vivo* may not always be accurately reflected in *ex vivo* suppression experiments (45). This notion is further supported by the finding that in a spontaneous melanoma model ROS-producing monocytic effector cells have direct antitumor activity that is independent of T cells (27). It is possible that in our model monocytes and moDCs exerted some direct antitumor activity via iNOS and TNFα, as these factors were expressed by monocytes and moDC in the dLN and also in the tumor (data not shown). However, MSU + *Msmeg* therapy of B16 melanoma was CD8^+^ T cell dependent (9), and CD8^+^ T cell proliferation was strongly diminished when moDCs were blocked. Therefore, one major role of moDCs was in supporting the priming of CD8^+^ T cells, although we cannot rule out additional direct antitumor functions.

We have previously shown that in the steady state CD11c^+^ DCs are necessary for crosspresentation to tumor-specific CD8^+^ T cells in dLN, as CD8^+^ T cell proliferation was abrogated in mice depleted of CD11c^+^ cells (9). The experiments presented here reveal that, during immunotherapy, blockade of CSF1R-dependent monocytes and moDCs reduced CD8^+^ T cell proliferation in dLN to a level similar to that observed in the steady state. Therefore, promoting the activation of adaptive antitumor immunity is likely to be a key function of moDCs during MSU + *Msmeg* therapy. We also observed a striking impact of moDCs on the infiltration of CD8^+^ T cells into tumors. This may merely reflect increased priming in dLN, or it may be due to increased recruitment of effector cells driven by monocytes and/or moDCs. It is also possible that moDCs may prime CD8^+^ T cells directly at the tumor site as suggested by Ma et al. (29), but the profound differences we see in dLN suggest that functionally relevant responses are occurring at this site during MSU + *Msmeg* therapy.

Transferred monocytes were sufficient to restore infiltration of proliferated CD8^+^ T cells into the tumors of CD11c-depleted mice. This finding suggests that moDCs can crossprime CD8^+^ T cells, either directly, or perhaps in cooperation with other cell subsets in CD11c-depleted mice. CD8^+^ or CD103^+^ DCs are known to be particularly effective at crosspriming, and two recent papers report that the presence of CD103^+^ DCs in tumors correlates with spontaneous tumor rejection (4) and response to treatment with paclitaxel in combination with CSF1 blockade (21). While those papers highlight the function of CD103^+^ DCs, they do not rule out a role for other DC subsets. Under physiological conditions, different DC subsets, such as CD103^+^ DCs and moDCs, may collaborate to induce optimal antitumor CD8^+^ T cell responses. The relative importance of both subsets may depend on the type of tumor, as different tumors vary in the ratio of CD103^+^ and CD11b^+^ DCs that infiltrate them (4), and would also be substantially affected by the inflammatory environment (9, 29). Furthermore, therapies may also preferentially activate CD103^+^ DCs or moDCs depending on the expression of receptors for damage- and pathogen-associated molecular patterns in these cell populations. A systematic study comparing the effect of different treatments on several DC subsets and in a range of tumors would be needed to fully address this issue.

CSF1-dependent monocytes and monocyte-derived cells including tumor-associated macrophages, MDSCs, and moDCs express a vast range of receptors to sense the local environment and are highly plastic in their response to various stimuli. This may explain why several studies show improved antitumor responses after CSF1/CSF1R blockade when combined with radiotherapy, chemotherapy, or antiangiogenic therapy (21, 37, 46–48) but no difference with CSF1 inhibition alone (46, 48, 49), or even suggest that monocytic cells may be important for antitumor responses (27, 29, 50). Here, we show that CSF1R inhibition can have a detrimental effect on tumor therapy, not only by depleting macrophage effector cells (51) but also by impairing the activation of tumor-specific T cells during immunotherapy. In line with the variable results of CSF1/CSF1R blockade, CSF1 gene signatures themselves were found to be either associated with poor survival or with improved survival, depending on the specific subtype of breast cancer (52). In a similar manner, macrophages can eliminate tumors through secretion of cytotoxic factors (44, 45) and be associated with increased survival in patients (53), but more often they promote tumor growth (21, 54). Even tumor-infiltrating MDSCs, generally thought to be highly immune suppressive, have been shown to exhibit plasticity and acquire the ability to crossprime antitumor CD8^+^ T cells in response to IL-12 (55).

CSF1R inhibitors are currently being tested in clinical trials of cancer therapy in patients^1^. However, our data indicate that in some cases CSF1 may actually be critical for the initiation of antitumor responses. This highlights the need for caution in combining CSF1R blockade with other therapies. The role of the immune system in mediating antitumor responses not only after immunotherapy but also in certain chemotherapies and radiotherapy is increasingly recognized. Therefore, it is important to fully delineate the role of monocytic cell populations in different types of tumors and treatments before carefully considering the situations in which a combination with CSF1 blockade may be beneficial. Our results indicate that, for the purpose of tumor therapy, blocking or eliminating monocytic cells will likely be less productive than appropriately activating them to harness their properties and promote powerful antitumor immune responses.

## Author Contributions

SK and FR conceived the study; SK and JY executed experiments; SK, JY, and FR designed and analyzed experiments; SK and FR wrote the paper; SK, JY, and FR commented on the manuscript and approved it for submission.

## Conflict of Interest Statement

The authors declare that the research was conducted in the absence of any commercial or financial relationships that could be construed as a potential conflict of interest.

## Footnotes

^1^clinicaltrials.gov. Clinical trials for RO5509554: identifiers NCT02323191 and NCT01494688. Clinical trials for PLX3397: identifiers NCT02071940, NCT02371369, NCT01596751, NCT02401815, NCT02390752, NCT01349049, NCT01349036, NCT01790503, NCT01525602, NCT01004861.

## Abbreviations

BM, bone marrow; CSF1, colony-stimulating factor-1; CSF1R, CSF1 receptor; DAPI, 6′-diamidino-2-phenylindole; DC, dendritic cell; dLN, draining LN; DT, diphtheria toxin; moDC, monocyte-derived DC; *Msmeg*, *Mycobacterium smegmatis*; MSU, monosodium urate; MDSC, myeloid-derived suppressor cell; NK, natural killer; OVA, ovalbumin; ROS, reactive oxygen species; WT, wild type.

## References

[1] Ataera H Hyde E Price KM Stoitzner P Ronchese F. Murine melanoma-infiltrating dendritic cells are defective in antigen presenting function regardless of the presence of CD4CD25 regulatory T cells. PLoS One (2011) 6:e17515. 10.1371/journal.pone.0017515 21390236 PMC3048402

[2] Stoitzner P Green LK Jung JY Price KM Atarea H Kivell B Inefficient presentation of tumor-derived antigen by tumor-infiltrating dendritic cells. Cancer Immunol Immunother (2008) 57:1665–73. 10.1007/s00262-008-0487-4 18311487 PMC11029823

[3] Stumbles PA Himbeck R Frelinger JA Collins EJ Lake RA Robinson BW. Cutting edge: tumor-specific CTL are constitutively cross-armed in draining lymph nodes and transiently disseminate to mediate tumor regression following systemic CD40 activation. J Immunol (2004) 173:5923–8. 10.4049/jimmunol.173.10.5923 15528325

[4] Broz ML Binnewies M Boldajipour B Nelson AE Pollack JL Erle DJ Dissecting the tumor myeloid compartment reveals rare activating antigen-presenting cells critical for T cell immunity. Cancer Cell (2014) 26:638–52. 10.1016/j.ccell.2014.09.007 25446897 PMC4254577

[5] Movassagh M Spatz A Davoust J Lebecque S Romero P Pittet M Selective accumulation of mature DC-Lamp+ dendritic cells in tumor sites is associated with efficient T-cell-mediated antitumor response and control of metastatic dissemination in melanoma. Cancer Res (2004) 64:2192–8. 10.1158/0008-5472.CAN-03-2969 15026362

[6] Aranda F Vacchelli E Obrist F Eggermont A Galon J Sautes-Fridman C Trial watch: toll-like receptor agonists in oncological indications. Oncoimmunology (2014) 3:e29179. 10.4161/onci.29179 25083332 PMC4091055

[7] Broomfield SA van der Most RG Prosser AC Mahendran S Tovey MG Smyth MJ Locally administered TLR7 agonists drive systemic antitumor immune responses that are enhanced by anti-CD40 immunotherapy. J Immunol (2009) 182:5217–24. 10.4049/jimmunol.0803826 19380767

[8] Kawarada Y Ganss R Garbi N Sacher T Arnold B Hammerling GJ. NK- and CD8(+) T cell-mediated eradication of established tumors by peritumoral injection of CpG-containing oligodeoxynucleotides. J Immunol (2001) 167:5247–53. 10.4049/jimmunol.167.9.5247 11673539

[9] Kuhn S Hyde EJ Yang J Rich FJ Harper JL Kirman JR Increased numbers of monocyte-derived dendritic cells during successful tumor immunotherapy with immune-activating agents. J Immunol (2013) 191:1984–92. 10.4049/jimmunol.1301135 23858033

[10] Vicari AP Chiodoni C Vaure C Ait-Yahia S Dercamp C Matsos F Reversal of tumor-induced dendritic cell paralysis by CpG immunostimulatory oligonucleotide and anti-interleukin 10 receptor antibody. J Exp Med (2002) 196:541–9. 10.1084/jem.20020732 12186845 PMC2196048

[11] Kuhn S Yang J Hyde E Harper JL Kirman JR Ronchese F. IL-1βR-dependent priming of anti-tumor CD4 T cells and sustained anti-tumor immunity after peri-tumoral treatment with MSU and mycobacteria. Oncoimmunology (2015) 4:e1042199. 10.1080/2162402X.2015.1042199 26451307 PMC4589042

[12] Rich FJ Kuhn S Hyde EJ Harper JL Ronchese F Kirman JR. Induction of T cell responses and recruitment of an inflammatory dendritic cell subset following tumor immunotherapy with *Mycobacterium smegmatis*. Cancer Immunol Immunother (2012) 61:2333–42. 10.1007/s00262-012-1291-8 22714285 PMC11042503

[13] Serbina NV Salazar-Mather TP Biron CA Kuziel WA Pamer EG. TNF/iNOS-producing dendritic cells mediate innate immune defense against bacterial infection. Immunity (2003) 19:59–70. 10.1016/S1074-7613(03)00171-7 12871639

[14] Le Borgne M Etchart N Goubier A Lira SA Sirard JC van Rooijen N Dendritic cells rapidly recruited into epithelial tissues via CCR6/CCL20 are responsible for CD8+ T cell crosspriming in vivo. Immunity (2006) 24:191–201. 10.1016/j.immuni.2006.01.005 16473831

[15] León B López-Bravo M Ardavín C. Monocyte-derived dendritic cells formed at the infection site control the induction of protective T helper 1 responses against *Leishmania*. Immunity (2007) 26:519–31. 10.1016/j.immuni.2007.01.017 17412618

[16] Rosas M Thomas B Stacey M Gordon S Taylor PR. The myeloid 7/4-antigen defines recently generated inflammatory macrophages and is synonymous with Ly-6B. J Leukoc Biol (2010) 88:169–80. 10.1189/jlb.0809548 20400676 PMC2892525

[17] Plantinga M Guilliams M Vanheerswynghels M Deswarte K Branco-Madeira F Toussaint W Conventional and monocyte-derived CD11b(+) dendritic cells initiate and maintain T helper 2 cell-mediated immunity to house dust mite allergen. Immunity (2013) 38:322–35. 10.1016/j.immuni.2012.10.016 23352232

[18] Goldszmid RS Caspar P Rivollier A White S Dzutsev A Hieny S NK cell-derived interferon-γ orchestrates cellular dynamics and the differentiation of monocytes into dendritic cells at the site of infection. Immunity (2012) 36:1047–59. 10.1016/j.immuni.2012.03.026 22749354 PMC3412151

[19] Nakano H Lin KL Yanagita M Charbonneau C Cook DN Kakiuchi T Blood-derived inflammatory dendritic cells in lymph nodes stimulate acute T helper type 1 immune responses. Nat Immunol (2009) 10:394–402. 10.1038/ni.1707 19252492 PMC2668134

[20] Braumüller H Wieder T Brenner E Aßmann S Hahn M Alkhaled M T-helper-1-cell cytokines drive cancer into senescence. Nature (2013) 494:361–5. 10.1038/nature11824 23376950

[21] Ruffell B Chang-Strachan D Chan V Rosenbusch A Ho CMT Pryer N Macrophage IL-10 blocks CD8(+) T cell-dependent responses to chemotherapy by suppressing IL-12 expression in intratumoral dendritic cells. Cancer Cell (2014) 26:623–37. 10.1016/j.ccell.2014.09.006 25446896 PMC4254570

[22] Movahedi K Laoui D Gysemans C Baeten M Stangé G Van den Bossche J Different tumor microenvironments contain functionally distinct subsets of macrophages derived from Ly6C(high) monocytes. Cancer Res (2010) 70:5728–39. 10.1158/0008-5472.CAN-09-4672 20570887

[23] Qian B-Z Li J Zhang H Kitamura T Zhang J Campion LR CCL2 recruits inflammatory monocytes to facilitate breast-tumour metastasis. Nature (2011) 475:222–5. 10.1038/nature10138 21654748 PMC3208506

[24] Franklin RA Liao W Sarkar A Kim MV Bivona MR Liu K The cellular and molecular origin of tumor-associated macrophages. Science (2014) 344:921–5. 10.1126/science.1252510 24812208 PMC4204732

[25] Noy R Pollard JW. Tumor-associated macrophages: from mechanisms to therapy. Immunity (2014) 41:49–61. 10.1016/j.immuni.2014.06.010 25035953 PMC4137410

[26] Khaled YS Ammori BJ Elkord E. Myeloid-derived suppressor cells in cancer: recent progress and prospects. Immunol Cell Biol (2013) 91:493–502. 10.1038/icb.2013.29 23797066

[27] Pommier A Audemard A Durand A Lengagne R Delpoux A Martin B Inflammatory monocytes are potent antitumor effectors controlled by regulatory CD4+ T cells. Proc Natl Acad Sci U S A (2013) 110:13085–90. 10.1073/pnas.1300314110 23878221 PMC3740849

[28] Stary G Bangert C Tauber M Strohal R Kopp T Stingl G. Tumoricidal activity of TLR7/8-activated inflammatory dendritic cells. J Exp Med (2007) 204:1441–51. 10.1084/jem.20070021 17535975 PMC2118597

[29] Ma Y Adjemian S Mattarollo SR Yamazaki T Aymeric L Yang H Anticancer chemotherapy-induced intratumoral recruitment and differentiation of antigen-presenting cells. Immunity (2013) 38:729–41. 10.1016/j.immuni.2013.03.003 23562161

[30] Ma Y Mattarollo SR Adjemian S Yang H Aymeric L Hannani D CCL2/CCR2-dependent recruitment of functional antigen-presenting cells into tumors upon chemotherapy. Cancer Res (2014) 74:436–45. 10.1158/0008-5472.CAN-13-1265 24302580

[31] Lugade AA Moran JP Gerber SA Rose RC Frelinger JG Lord EM. Local radiation therapy of B16 melanoma tumors increases the generation of tumor antigen-specific effector cells that traffic to the tumor. J Immunol (2005) 174:7516–23. 10.4049/jimmunol.174.12.7516 15944250

[32] Ma JZ-I Lim SN Qin JS Yang J Enomoto N Ruedl C Murine CD4+ T cell responses are inhibited by cytotoxic T cell-mediated killing of dendritic cells and are restored by antigen transfer. PLoS One (2012) 7:e37481. 10.1371/journal.pone.0037481 22649530 PMC3359309

[33] Conway JG McDonald B Parham J Keith B Rusnak DW Shaw E Inhibition of colony-stimulating-factor-1 signaling in vivo with the orally bioavailable cFMS kinase inhibitor GW2580. Proc Natl Acad Sci U S A (2005) 102:16078–83. 10.1073/pnas.0502000102 16249345 PMC1276040

[34] Serbina NV Pamer EG. Monocyte emigration from bone marrow during bacterial infection requires signals mediated by chemokine receptor CCR2. Nat Immunol (2006) 7:311–7. 10.1038/ni1309 16462739

[35] Geissmann F Jung S Littman DR. Blood monocytes consist of two principal subsets with distinct migratory properties. Immunity (2003) 19:71–82. 10.1016/S1074-7613(03)00174-2 12871640

[36] Getts DR Terry RL Getts MT Müller M Rana S Shrestha B Ly6c+ “inflammatory monocytes” are microglial precursors recruited in a pathogenic manner in West Nile virus encephalitis. J Exp Med (2008) 205:2319–37. 10.1084/jem.20080421 18779347 PMC2556789

[37] Mitchem JB Brennan DJ Knolhoff BL Belt BA Zhu Y Sanford DE Targeting tumor-infiltrating macrophages decreases tumor-initiating cells, relieves immunosuppression, and improves chemotherapeutic responses. Cancer Res (2013) 73:1128–41. 10.1158/0008-5472.CAN-12-2731 23221383 PMC3563931

[38] Segura E Albiston AL Wicks IP Chai SY Villadangos JA. Different cross-presentation pathways in steady-state and inflammatory dendritic cells. Proc Natl Acad Sci U S A (2009) 106:20377–81. 10.1073/pnas.0910295106 19918052 PMC2787113

[39] Cheong C Matos I Choi JH Dandamudi DB Shrestha E Longhi MP Microbial stimulation fully differentiates monocytes to DC-SIGN/CD209(+) dendritic cells for immune T cell areas. Cell (2010) 143:416–29. 10.1016/j.cell.2010.09.039 21029863 PMC3150728

[40] Greter M Helft J Chow A Hashimoto D Mortha A Agudo-Cantero J GM-CSF controls nonlymphoid tissue dendritic cell homeostasis but is dispensable for the differentiation of inflammatory dendritic cells. Immunity (2012) 36:1031–46. 10.1016/j.immuni.2012.03.027 22749353 PMC3498051

[41] Shime H Matsumoto M Oshiumi H Tanaka S Nakane A Iwakura Y Toll-like receptor 3 signaling converts tumor-supporting myeloid cells to tumoricidal effectors. Proc Natl Acad Sci U S A (2012) 109:2066–71. 10.1073/pnas.1113099109 22308357 PMC3277567

[42] Singer K Gottfried E Kreutz M Mackensen A. Suppression of T-cell responses by tumor metabolites. Cancer Immunol Immunother (2011) 60:425–31. 10.1007/s00262-010-0967-1 21240484 PMC11029601

[43] Chang CI Liao JC Kuo L. Macrophage arginase promotes tumor cell growth and suppresses nitric oxide-mediated tumor cytotoxicity. Cancer Res (2001) 61:1100–6. 11221839

[44] Brantley EC Guo L Zhang C Lin Q Yokoi K Langley RR Nitric oxide-mediated tumoricidal activity of murine microglial cells. Transl Oncol (2010) 3:380–8. 10.1593/tlo.10208 21151477 PMC3000463

[45] Miguel RDV Cherpes TL Watson LJ McKenna KC. CTL induction of tumoricidal nitric oxide production by intratumoral macrophages is critical for tumor elimination. J Immunol (2010) 185:6706–18. 10.4049/jimmunol.0903411 21041723 PMC3152256

[46] Priceman SJ Sung JL Shaposhnik Z Burton JB Torres-Collado AX Moughon DL Targeting distinct tumor-infiltrating myeloid cells by inhibiting CSF-1 receptor: combating tumor evasion of antiangiogenic therapy. Blood (2010) 115:1461–71. 10.1182/blood-2009-08-237412 20008303 PMC2826767

[47] Ries CH Cannarile MA Hoves S Benz J Wartha K Runza V Targeting tumor-associated macrophages with anti-CSF-1R antibody reveals a strategy for cancer therapy. Cancer Cell (2014) 25:846–59. 10.1016/j.ccr.2014.05.016 24898549

[48] Xu J Escamilla J Mok S David J Priceman S West B CSF1R signaling blockade stanches tumor-infiltrating myeloid cells and improves the efficacy of radiotherapy in prostate cancer. Cancer Res (2013) 73:2782–94. 10.1158/0008-5472.CAN-12-3981 23418320 PMC4097014

[49] MacDonald KPA Palmer JS Cronau S Seppanen E Olver S Raffelt NC An antibody against the colony-stimulating factor 1 receptor depletes the resident subset of monocytes and tissue- and tumor-associated macrophages but does not inhibit inflammation. Blood (2010) 116:3955–63. 10.1182/blood-2010-02-266296 20682855

[50] Li M Knight DA A Snyder L. A role for CCL2 in both tumor progression and immunosurveillance. Oncoimmunology (2013) 2:e25474. 10.4161/onci.25474 24073384 PMC3782157

[51] van der Sluis TC Sluijter M van Duikeren S West BL Melief CJ Arens R Therapeutic peptide vaccine-induced CD8 T cells strongly modulate intratumoral macrophages required for tumor regression. Cancer Immunol Res (2015) 3:1042–51. 10.1158/2326-6066.CIR-15-0052 25888578

[52] Beck AH Espinosa I Edris B Li R Montgomery K Zhu S The macrophage colony-stimulating factor 1 response signature in breast carcinoma. Clin Cancer Res (2009) 15:778–87. 10.1158/1078-0432.CCR-08-1283 19188147 PMC2987696

[53] Ohri CM Shikotra A Green RH Waller DA Bradding P. Macrophages within NSCLC tumour islets are predominantly of a cytotoxic M1 phenotype associated with extended survival. Eur Respir J (2009) 33:118–26. 10.1183/09031936.00065708 19118225

[54] Qian B-Z Pollard JW. Macrophage diversity enhances tumor progression and metastasis. Cell (2010) 141:39–51. 10.1016/j.cell.2010.03.014 20371344 PMC4994190

[55] Kerkar SP Goldszmid RS Muranski P Chinnasamy D Yu Z Reger RN IL-12 triggers a programmatic change in dysfunctional myeloid-derived cells within mouse tumors. J Clin Invest (2011) 121:4746–57. 10.1172/JCI58814 22056381 PMC3226001

