# Supplementary material for: Monocyte-Derived Dendritic Cells Are Essential for CD8+ T Cell Activation and Antitumor Responses After Local Immunotherapy
Source: Front Immunol. 2015 Nov 23;6:584. doi: 10.3389/fimmu.2015.00584 (PMC4655312; doi:10.3389/fimmu.2015.00584)
Supplement: Supplementary file 1 [file Data_Sheet_1.PDF]

Kuhn et al, **Monocyte-derived dendritic cells are essential for CD8$^{+}$ T cell activation and anti-tumor responses after local immunotherapy**

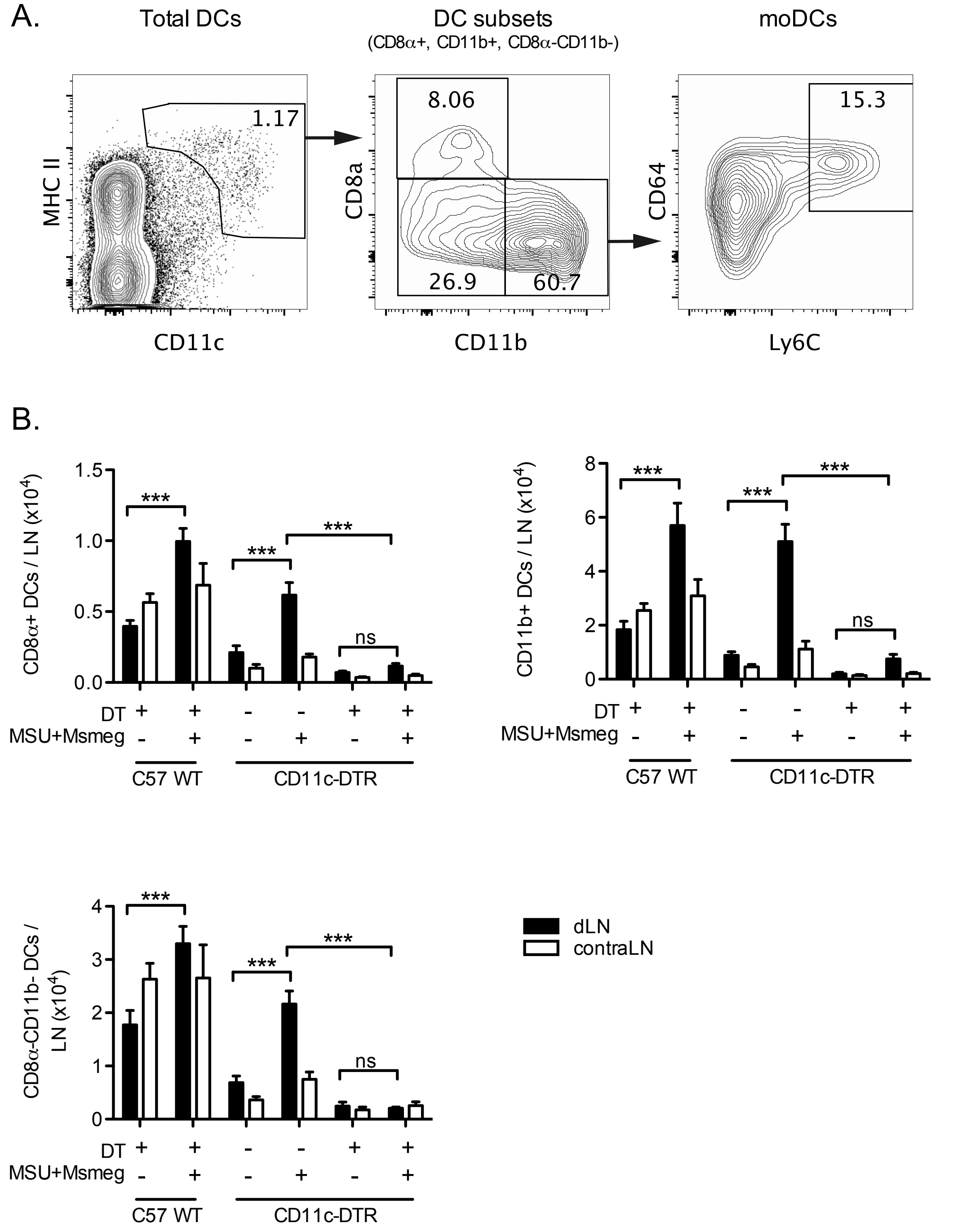


**Figure S1: Gating of DC subsets in LNs and DC subset depletion in CD11c-DTR BM chimeras.**

C57BL/6 WT (C57WT) or CD11c-DTR BM chimeras were injected with B16.OVA melanoma tumors, treated every second day for 4 times with MSU+Msmeg or PBS, and at the same time depleted of CD11c+ cells by DT treatment as indicated. Two

days after the 4th MSU+Msmeg treatment, mice were sacrificed and LN were examined for DC depletion by flow cytometry. (A) Gating strategy. After pre-gating on live single cells, DCs were identified as MHCII+ CD11c+, and further subdivided into CD8α+, CD11b+, or CD8α-CD11b- subsets. Ly6C+CD64+ moDCs were identified within the CD11b+ DC subset. (B) Numbers of DCs in the CD8α+, CD11b+, or CD8α-CD11b- subsets are shown as mean + SEM for 5 mice/group. Statistical analysis was by one-way-ANOVA with Tukey's post test.
